# Experimental Study on Mechanical Properties and Permeability Characteristics of Calcareous Mudstone under Different Confining Pressures

**DOI:** 10.3390/ma17112731

**Published:** 2024-06-04

**Authors:** Wei Xu, Xiao Qu, Long Yan, Yu Ning

**Affiliations:** 1 Research Institute of Geotechnical Engineering, Hohai University, Nanjing 210098, China; weixuhhu@163.com (W.X.); yanlong@hhu.edu.cn (L.Y.); 2 College of Energy and Mining Engineering, Shandong University of Science and Technology, Qingdao 266590, China; 3 University of Lille, French National Centre for Scientific Research, Centrale Lille, LaMcube, 59000 Lille, France; 4 PowerChina Kunming Engineering Corporation Limited, Kunming 650035, China; gladningyu@gmail.com

**Keywords:** calcareous mudstone, mechanical properties, permeability characteristics, energy evolution, crack propagation

## Abstract

Calcareous mudstone, a type of red-bed soft rock, is prevalent in the surrounding rock of the Central Yunnan Water Diversion Project (CYWDP) in Yunnan Province, China, significantly impacting both construction and operation. The mechanical properties of calcareous mudstone vary with depth. This study investigates its mechanical properties, permeability characteristics, energy evolution, and macro- and micro-failure characteristics during deformation using triaxial compression tests under different confining pressures. Results reveal distinct stage characteristics in the stress–strain behavior, permeability, and energy evolution of calcareous mudstone. Crack propagation, permeability evolution, and energy dissipation are closely linked, elucidating the deformation and failure process, with fluid pressure playing a crucial role. The confining pressure *σ*_3_ increased from 2 MPa to 4 MPa and 6 MPa, while the peak stress *σ*_c_ (*P*_w_ = 1 MPa) of the calcareous mudstone increased by 84.49% and 24.89%, respectively. Conversely, the permeability at *σ*_c_ decreased from 11.25 × 10^−17^ m^2^ to 8.99 × 10^−17^ m^2^ and 5.72 × 10^−17^ m^2^, while the dissipative energy at *σ*_c_ increased from 12.39 kJ/m^3^ to 21.14 kJ/m^3^ and 42.51 kJ/m^3^. In comparison to those without fluid pressure (*P*_w_ = 0), the value of *σ*_c_ at *P*_w_ = 1 MPa was reduced by 36.61%, 23.23%, and 20.67% when *σ*_3_ was 2, 4, and 6 MPa, respectively. Increasing confining pressure augments characteristic stresses, deformation and failure energy, and ductility, while reducing permeability, crack propagation, and width. These findings enhance our understanding of calcareous mudstone properties at varying depths in tunnel construction scenarios.

## 1. Introduction

It is generally recognized that rock plays a vital role in various engineering fields, such as hydropower engineering [1], tunnel engineering [2], energy storage [3], and nuclear waste disposal engineering [4]. Recently, there has been a surge in engineering projects conducted in deep underground environments, which pose significant challenges due to their complexity. The rock mass is subjected to high temperatures and water, which generate equivalent forces that can result in a reduction in its bearing and deformation capacity [5]. The mechanical and permeability properties of rock are known to vary depending on the prevailing stress conditions [6]. Crack initiation, propagation, energy accumulation, conversion, and rock deformation and failure are progressive processes. Consequently, the mechanical and permeability properties of rock undergo dynamic evolution. Therefore, investigating the mechanical behavior, permeability, and energy characteristics of rock under different stress states can offer valuable insights into the internal mechanism of rock failure, thus ensuring engineering stability and safety.

It is imperative to analyze characteristic stress thresholds and the progressive failure process for rock mass excavation and construction in engineering. For instance, the peak stress serves as a crucial project parameter, the damage stress guides design decisions in determining the project’s long-term strength, and the initiation stress helps analyze phenomena like spalling in tunnel surrounding rock [7,8,9]. Numerous scholars have focused on researching characteristic stress thresholds, deformation, and the failure process of rock [6,8,10,11]. Primary research methods include the crack volumetric strain method (CVSM) [7,10,12,13], the acoustic emission method [14,15], and the moving-point regression method [16,17]. Seepage pressure significantly influences the mechanical behavior of rocks in engineering. Therefore, investigating the seepage characteristics of rock under various stress environments is essential for engineering construction. The interaction of water–rock coupling typically occurs when groundwater is present. Changes in rock structure due to excavation disturbance can affect rock permeability. Numerous studies have demonstrated that rock permeability is influenced by factors such as confining pressure [18], stress [19], strain [20], fluid pressure [21], microfracture structure [22], temperature [23] and fluid [24]. Rock deformation and failure are strongly related to the propagation of internal fractures [25,26,27], contributing significantly to the field of rock permeability. However, previous investigations have predominantly focused on hard or relatively hard rock, leaving the permeability evolution law of soft rock during compressive failure to be further explored.

During deformation and failure processes, rocks experience energy transfer and conversion [28,29]. Recent studies have focused on investigating the mechanical properties and energy variations in various rock types, including marble [30], basalt [31], granite [32], coal [33], phyllite [34], sandstone [35], among others, employing energy theories. For instance, Zhang et al. [36] explored the mechanical and evolution characteristics of coal in various depths using triaxial compression tests. Chen et al. [37] investigated the mechanical behavior of six rock types through uniaxial and triaxial compression tests, examining the influence of lithology and confining pressure on rock energy evolution. Xia et al. [38] conducted experiments on sandstone using acoustic emission and uniaxial compression to analyze energy variations. However, there is a lack of sufficient research elucidating the internal relationship between crack propagation and permeability characteristics in soft rocks, as well as the mechanism of energy evolution following water-induced softening.

This study performs triaxial compression tests on calcareous mudstone under various confining pressures in both its natural state and under fluid pressure. The investigation aims to analyze characteristic stresses, crack propagation, permeability, energy evolution, and failure characteristics during deformation and failure processes. Furthermore, it explores the impact of confining pressure on these mechanical properties. Beginning with an examination of the water–rock interaction of calcareous mudstone, this study elucidates the mechanisms underlying its deformation, failure, permeability, and energy evolution. The findings of this study can offer valuable guidance and evaluation for the construction and operation of practical projects.

## 2. Experimental Investigations

### 2.1. Description of Specimen

**Figure 1:**
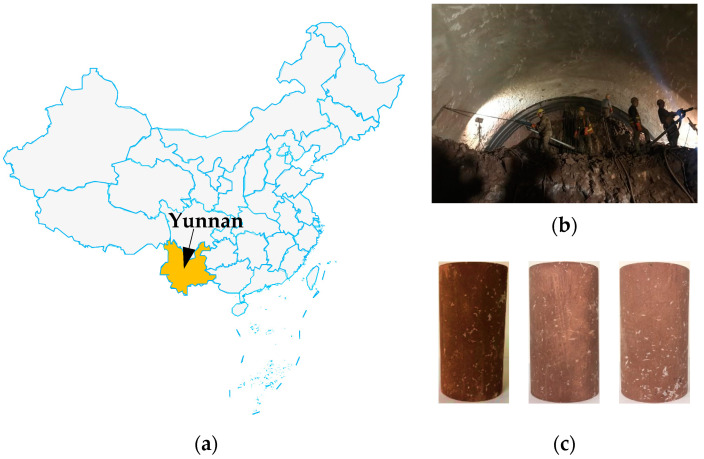
Sampling area and typical test specimens of calcareous mudstone: (**a**) location of the CYWDP; (**b**) tunnel site; (**c**) test specimens.

The length of the CYWDP spans 661.77 km, with 208.49 km (31.5%) consisting of soft rock tunnels, predominantly composed of red-bed soft rock. Calcareous mudstone blocks were collected from the diversion tunnels (see Figure 1a,b). The calcareous mudstone has weakly intercalated calcium layers or structural planes, along with quartz, feldspar, and calcite, alongside a notable presence of water-sensitive clay minerals. The cylindrical specimens, measuring 50 mm × 100 mm and processed from the same rock block, exhibited a red coloration with no visible cracks, as depicted in Figure 1c.

**Figure 2:**
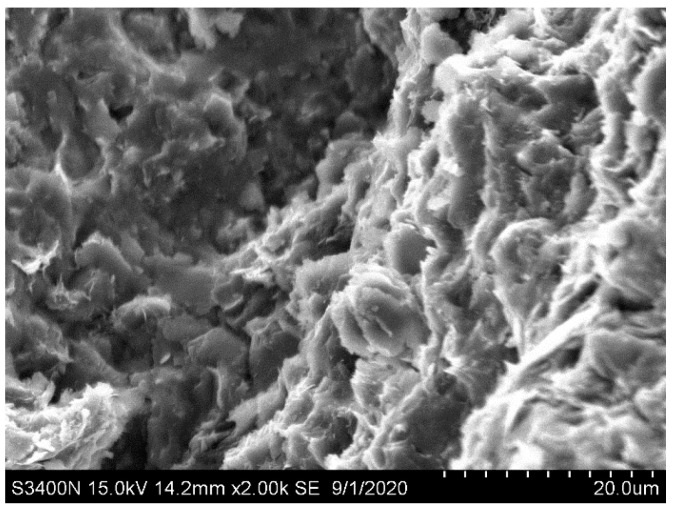
Microstructure of a rock specimen shown by SEM at 2000 times magnification.

The microstructural surface morphology of the calcareous mudstone was examined using scanning electron microscopy (SEM) at a magnification of 2000 times (Figure 2). The micrograph reveals that the calcareous mudstone exhibits predominantly high cementation, with a prevalent mixed-layer structure (e.g., flaky, blocky). The fracture morphology displays irregularities, characterized by a mixture of crystalline grains and clay minerals, with numerous irregularly shaped grains observed along the fracture surfaces.

### 2.2. Test Apparatus and Methods

**Figure 3:**
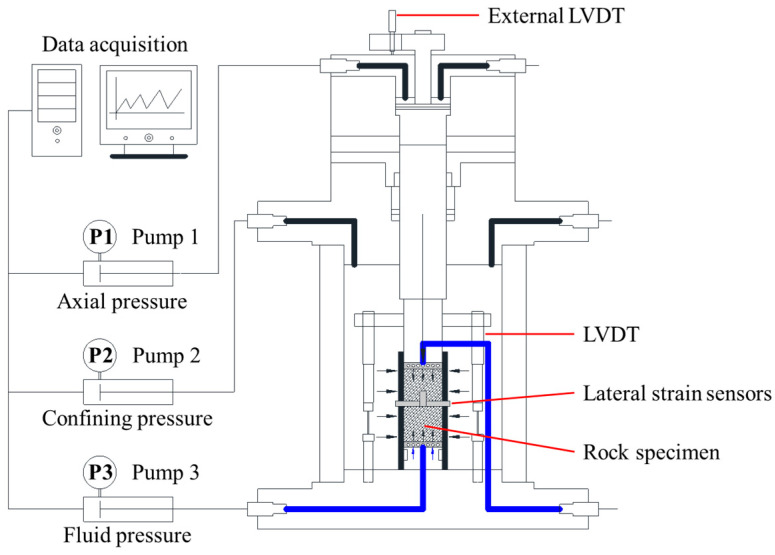
Triaxial test system.

The rock rheology triaxial system was employed to conduct triaxial compression tests on calcareous mudstone under varying confining pressures. The test system, as detailed by Wang et al. [39,40], features three high-precision pumps for supplying deviatoric stress, confining pressure, and fluid pressure, as illustrated in Figure 3.

To investigate the mechanical behavior and permeability of calcareous mudstone, two test protocols [41] were devised based on the geological conditions of the diversion project, in situ stress, and groundwater distribution. For each protocol, three rock specimens were selected:

1. Triaxial compression tests without fluid pressure: Natural calcareous mudstone specimens underwent triaxial compression tests at confining pressures of 2, 4, and 6 MPa. First, a natural calcareous mudstone specimen was installed in a triaxial pressure cell. The sensors were then adjusted, and the confining pressure was subsequently applied at a rate of 0.5 MPa/s to the desired value. Once the confining pressure had reached a stable state, axial loading was applied at a rate of 0.02 mm/min until the specimen failed.
2. Triaxial compression tests incorporating fluid pressure: Calcareous mudstone specimens were subjected to triaxial compression tests under a fluid pressure of 1 MPa and confining pressures of 2, 4, and 6 MPa. It was essential to evacuate and saturate the specimen before testing. The procedure followed the aforementioned steps until the confining pressure reached a stable state. Subsequently, fluid pressure was applied at 1 MPa and allowed to stabilize. Thereafter, axial loading was conducted at a rate of 0.02 mm/min until the specimen failed. The permeability of calcareous mudstone was evaluated using a steady-state approach [21].

## 3. Test Results

### 3.1. Stress–Strain Relationships and Crack Propagation Laws

**Figure 4:**
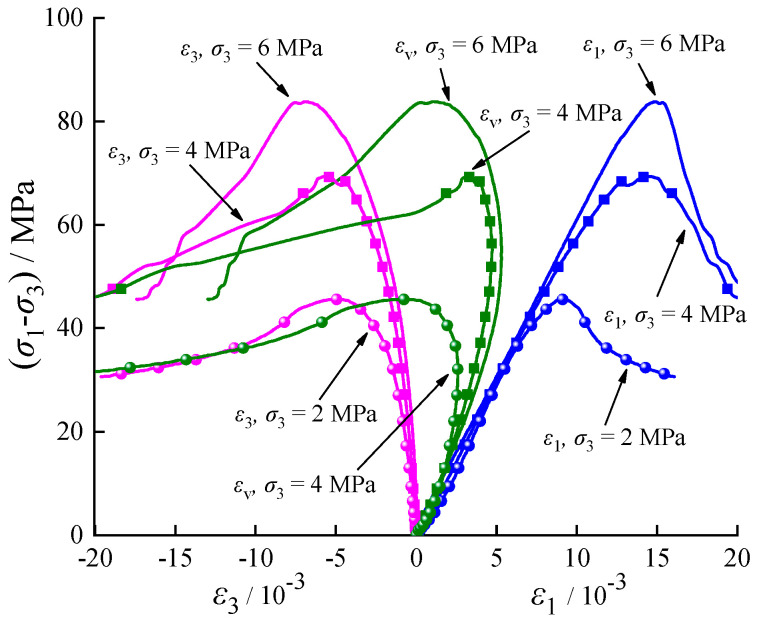
Stress-strain relationships.

**Figure 5:**
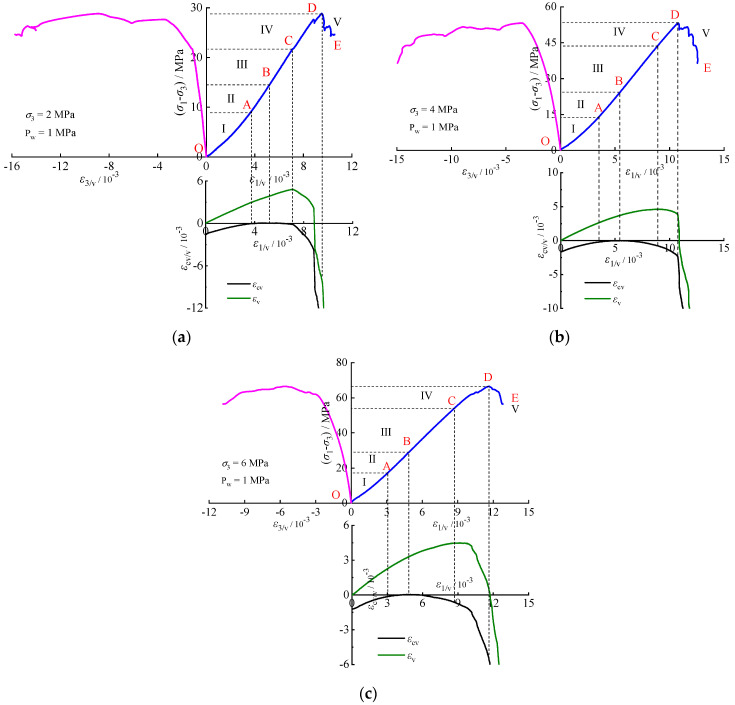
Stress–strain relationships at 1 MPa fluid pressure: (**a**) $\sigma_{3}=2$ MPa; (**b**) $\sigma_{3}=4$ MPa; (**c**) $\sigma_{3}=6$ MPa.

Table 1 presents the test results of rock specimens under various fluid pressures. The stress–strain relationships are shown in Figure 4 and Figure 5 (with fluid pressure *P*_w_ = 1 MPa), where *ε*_1_, *ε*_3_, *ε*_v_, and *ε*_cv_ represent the axial, lateral, volumetric, and crack volumetric strains, respectively, and (*σ*_1_ − *σ*_3_) denotes the deviatoric stress.

Table 1 and Figure 4 and Figure 5 illustrate that the crack damage stress, peak stress, and axial peak strain all increase with the confining pressure. Specifically, the peak stresses (with *P*_w_ = 0 and *P*_w_ = 1 MPa) of the calcareous mudstone increase by 52.35%, 20.85%, and 84.49%, 24.89%, respectively, at confining pressure of 2 MPa, 4 MPa, and 6 MPa. This suggests an enhancement in peak strength due to the increased confining pressure. Additionally, the damage stresses increase by 75.79%, 2.94%, and 98.53%, 25.56%, respectively, indicating that fluid pressure accelerates crack propagation. Furthermore, it is noteworthy that the rock’s ductility increases at higher confining pressures, as evidenced by the greater axial strain at which it reaches its peak stress.

Crack initiation and development serve as indicators of rock deformation and failure. Understanding progressive failure processes requires examining characteristic stresses. In this study, the characteristic stresses of the stress–strain curve (the upper limit stress of each stage, corresponding to points A, B, and C in Figure 5) were determined using the CVSM [7]. The stress–strain relationship for rock can be divided into five stages.

Stage Ⅰ (OA) represents crack compression and closure. In this stage, the crack volumetric strain moves in a positive direction, leading to the compression and closure of existing microcracks within the rock. This process is influenced by factors such as the number and morphology of existing cracks and stress conditions. The characteristic stress of this stage is the crack closure stress *σ*_cc_.

Stage Ⅱ (AB) corresponds to linear elastic deformation. During this stage, there is no sliding between cracks in the rock, and no new cracks form. The crack’s volumetric strain remains constant, resulting in a linear stress–strain relationship with a characteristic stress of the initiation stress *σ*_ci_.

Stage Ⅲ (BC) signifies stable crack propagation. Here, the crack volumetric strain shifts to the negative direction, indicating the formation and stable development of new cracks. Plastic deformation and volumetric dilatation occur in the rock, with characteristic stress being the damage stress *σ*_cd_.

Stage Ⅳ (CD) denotes unstable crack propagation. Microcracks rapidly propagate and connect, leading to accelerated dilatation of the rock volume. The characteristic stress at this stage is the peak stress *σ*_c_.

Stage Ⅴ (DE) represents the post-peak failure stage, where cracks in the rock interconnect to form a macro-failure plane, resulting in macro-failure of the rock specimen.

To explore the relationships between characteristic stresses and confining pressures, the effective stress principle [42] is considered when fluid pressure is involved. The effective confining pressure $P_{f}$ can be expressed as:

(1) $$P_{f}=\sigma_{3}-P_{w}$$

where $P_{f}$ is the effective confining pressure, $\sigma_{3}$ is the confining pressure, and $P_{w}$ is the fluid pressure.

**Figure 6:**
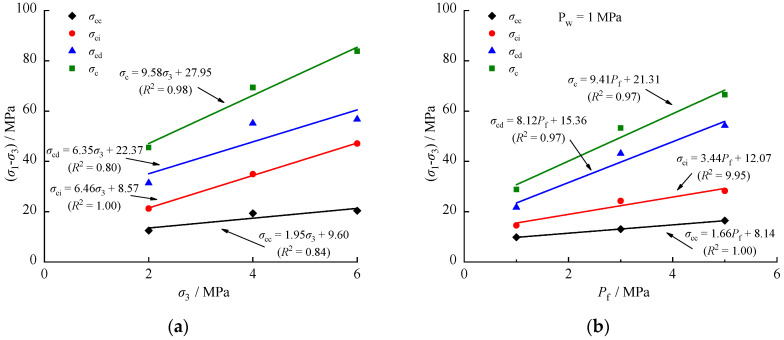
Relationships between characteristic stress and confining pressure: (**a**) *P*_w_ = 0; (**b**) *P*_w_ = 1 MPa.

Figure 6 illustrates the relationships between characteristic stresses and confining pressure $\sigma_{3}$, as well as effective confining pressure $P_{f}$. The ratios of *σ*_cc_, *σ*_ci_, and *σ*_cd_ to *σ*_c_ range from 24–34%, 42–47%, and 68–82%, respectively. The values of *σ*_cc_, *σ*_ci_, *σ*_cd_, and *σ*_c_ increase linearly with confining pressure. These results indicate that confining pressure effectively restricts the lateral propagation of micro- and macro-cracks. Additionally, the degree of compressional closure of the original cracks in the rock increases with higher confining pressure, resulting in higher density and strength. However, the characteristic stress of each stage under the same confining pressure is reduced by the presence of fluid pressure. For example, at a confining pressure of 2 MPa, the values of *σ*_cc_, *σ*_ci_, *σ*_cd_, and *σ*_c_ are reduced by 21.60%, 31.37%, 30.70%, and 36.61%, respectively, due to the effect of a fluid pressure of 1 MPa. This suggests that the mechanical properties of the rock are weakened by the fluid pressure.

### 3.2. Permeability Characteristics

**Figure 7:**
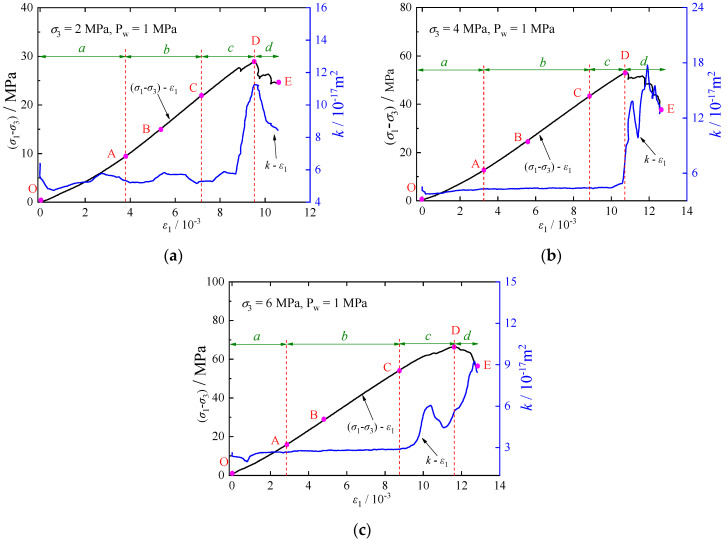
Evolutions of permeability-axial strain: (**a**) $\sigma_{3}=2$ MPa; (**b**) $\sigma_{3}=4$ MPa; (**c**) $\sigma_{3}=6$ MPa.

The permeability of rock can be influenced by changes in its microstructure. Figure 7 illustrates the evolution of permeability *k* with axial strain *ε*_1_ for calcareous mudstone at a fluid pressure of 1 MPa and confining pressures of 2 MPa, 4 MPa, and 6 MPa. It should be noted that this section determines the characteristic stresses and their location on the stress-strain curve based on the method described in Section 3.1. The relationship between permeability and axial strain is evident. The permeability of rock specimens under various confining pressures exhibits distinct nonlinear and staged features with axial strain, which are described by the following four phases.

Phase *a*: corresponds to Stage I (OA) in Section 3.1. With increasing axial strain, original cracks and pores of the rock are closed by compression, leading to a gradual decrease in permeability and the blocking of seepage paths.

Phase *b*: corresponds to Stage Ⅱ (AB) and Stage Ⅲ (BC), showing a small change in permeability. It is noteworthy that microcracks in the rock continue to be compressed and closed, and the volume decreases at a very low rate in Stage AB. Upon entering Stage BC, new microcracks begin to form, accompanied by volume dilatation.

Phase *c*: corresponds to Stage Ⅳ (CD), during which microcracks start to grow and coalesce, opening seepage paths and resulting in a significant increase in permeability. Interestingly, the peak stress corresponds to a permeability that is not at its maximum (the peak permeability lags behind the peak stress). As a result of the propagation and evolution of microcracks, the connectivity of the cracks (seepage paths) is enhanced, leading to an increase in permeability and a “jumping” phenomenon at this stage. This observation is consistent with the findings of previous studies [21,39].

Phase *d*: corresponds to Stage Ⅴ (DE), whereupon reaching the peak stress, the permeability increases rapidly to its peak due to the onset of macro-fracture plane. However, the permeability decreases as frictional sliding of the cracks progresses.

**Figure 8:**
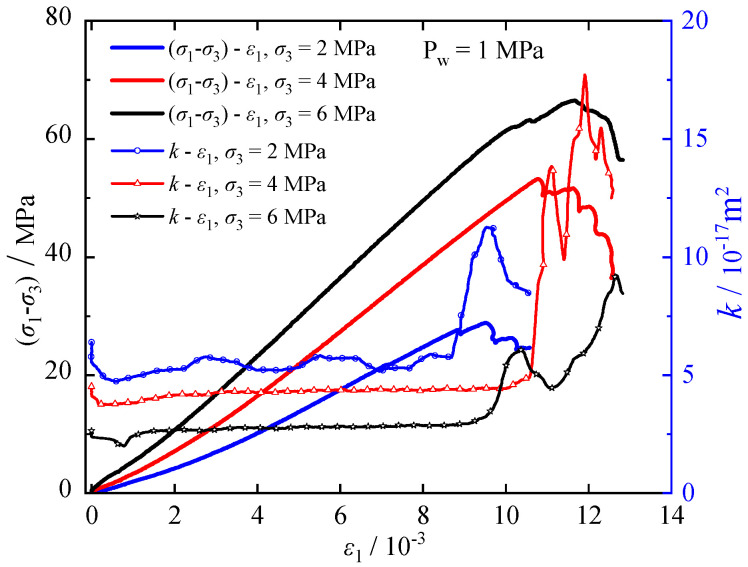
Relationships of stress, permeability and axial strain.

**Figure 9:**
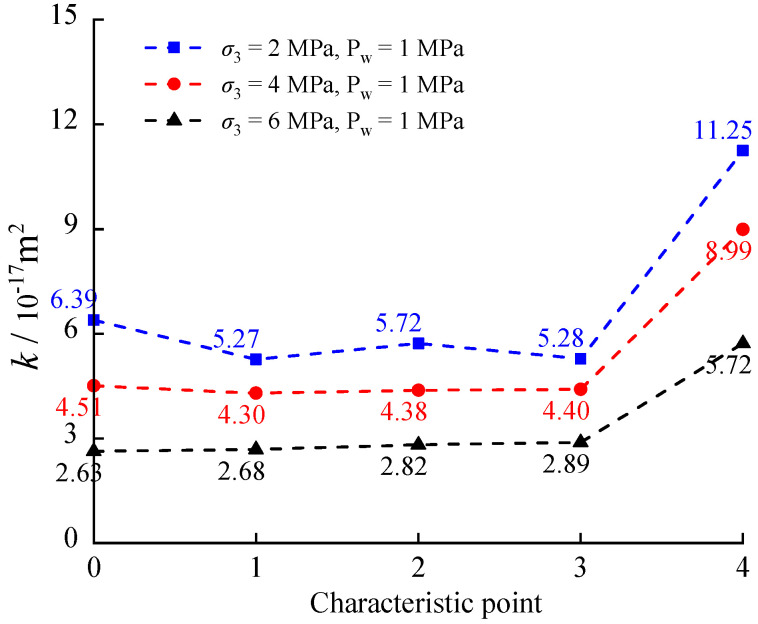
Comparison of permeability at stress characteristic values.

The relation of deviatoric stress ($\sigma_{1}-\sigma_{3}$), permeability *k*, and axial strain *ε*_1_ for a calcareous mudstone under 1 MPa fluid pressure at various confining pressures is shown in Figure 8. Furthermore, Figure 9 compares permeability *k* under characteristic stresses at different confining pressures. The horizontal coordinates 0, 1, 2, 3, and 4 represent the initial permeability *k*_0_ and the corresponding permeability at the crack closure stress *σ*_cc_, initiation stress *σ*_ci_, damage stress *σ*_cd_, and peak stress *σ*_c_, respectively. Figure 8 and Figure 9 depict a reduction in specimen permeability during the stage of crack compression closure. With an increase in confining pressure from 2 MPa to 4 MPa and 6 MPa, both the initial permeability and permeability during this stage exhibit decreasing trends. Elevated confining pressure compresses and seals pre-existing cracks and pores in the rock, intensifying friction and cohesion between rock grains, thereby restricting seepage paths and limiting water flow. During the phase of elastic deformation and crack stabilization, microcracks remain dormant, impeding the formation of seepage paths and resulting in minimal changes in permeability. However, upon reaching the stress damage threshold, cracks begin to merge and propagate, establishing seepage pathways and leading to a substantial increase in permeability. Despite the increase in confining pressure, the rise in permeability remains constrained at this stage.

**Figure 10:**
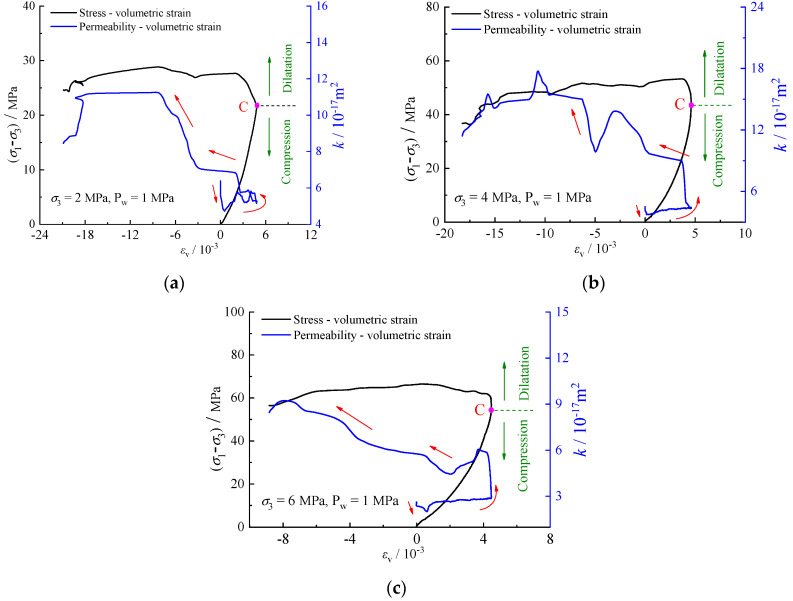
Evolution of permeability-volumetric strain: (**a**) $\sigma_{3}=2$ MPa; (**b**) $\sigma_{3}=4$ MPa; (**c**) $\sigma_{3}=6$ MPa.

Volumetric strain can influence the initiation and growth of microcracks, consequently affecting rock permeability. Figure 10 shows that the relationship between volumetric strain *ε*_v_ and permeability *k* of specimens under varying confining pressures. Permeability exhibits similar changes with volumetric strain. During the stage of crack compression and closure, microcrack closure results in a decrease in permeability alongside an increase in volumetric strain. Throughout the elastic deformation and stable crack propagation stages, permeability remains relatively constant while volumetric strain continues to increases. The trend of the volumetric strain curve shifts at point C, indicating a transition in the rock’s behavior from compression to dilation. Prior to point C, permeability decreases and stabilizes during the compression phase. Following point C, further propagation of microcracks leads to the formation of seepage paths, resulting in an increase in permeability and a shift towards dilation strain. The increase in confining pressure corresponds to an elevation in *σ*_cd_, suggesting that the rock’s deformation is constrained by the applied confining pressure.

### 3.3. Mechanism of Energy Evolution

Energy transfer and conversion play crucial roles in rock deformation and failure processes. Energy dissipation provides insights into crack initiation, damage accumulation, plastic deformation, and the gradual loss of strength leading to ultimate failure of the rock. In this section, the total energy during experimental loading, neglecting energy loss, is expressed as:

(2) $$U=U_{d}+U_{e}$$

where U represents the total energy, $U_{d}$ denotes dissipative energy, and $U_{e}$ stands for elastic strain energy.

During the loading of a conventional triaxial compression test ($\sigma_{2}=\sigma_{3}$), U and the $U_{e}$ are given by:

(3) $$U=U_{1}+U_{3}=\int_{0}^{\varepsilon_{1}}\sigma_{1}d\varepsilon_{1}+2\int_{0}^{\varepsilon_{3}}\sigma_{3}d\varepsilon_{3}$$

(4) $$U_{e}=\frac{1}{2E}\left[\sigma_{1}^{2}+2\sigma_{3}^{2}-2\upsilon \sigma_{3}(2\sigma_{1}+\sigma_{3})\right]$$

where $U_{1}$ represents the strain energy converted by the positive work done by $\sigma_{1}$, $U_{3}$ denotes the strain energy released by the negative work done by $\sigma_{3}$, E is the elastic modulus, and υ is Poisson’s ratio.

**Figure 11:**
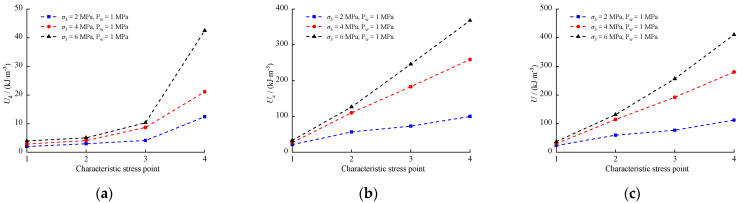
Comparison of energy values at characteristic stress points: (**a**) $U_{d}$; (**b**) $U_{e}$; (**c**) U.

Energy continuously converts and balances during rock loading and failure. A Comparison of the energy values at characteristic stresses during the failure process of calcareous mudstone is shown in Figure 11, where horizontal coordinates 1, 2, 3, and 4 represent the corresponding energies $U_{d}$, $U_{e}$, and U at the characteristic stresses $\sigma_{cc}$, $\sigma_{ci}$, $\sigma_{cd}$, and $\sigma_{c}$, respectively. It is observed that with increasing stress, $U_{d}$, $U_{e}$, and U increase at different rates at characteristic stresses of the rock, indicating that specific energy states correspond to specific stress states during stress increments. Moreover, $U_{d}$, $U_{e}$, and U exhibit varied increases as confining pressure rises for the same stress state, signifying that higher confining pressures demand more energy for rock deformation and failure.

**Figure 12:**
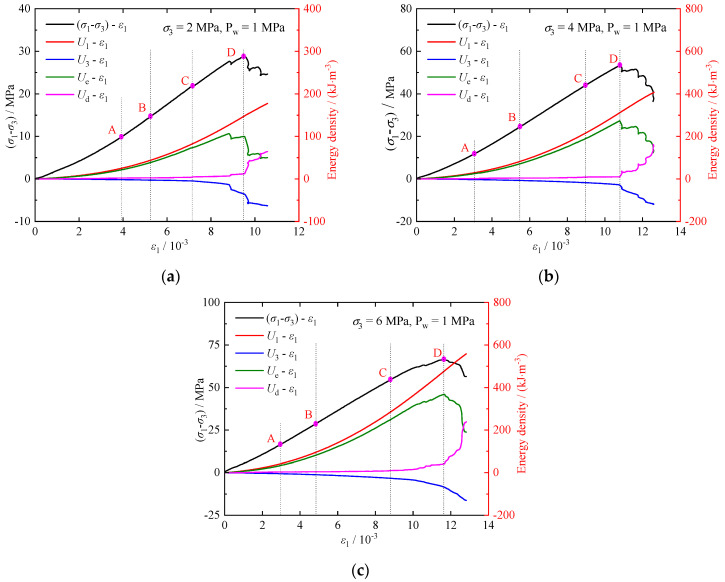
Evolutions of energy-axial strain: (**a**) $\sigma_{3}=2$ MPa; (**b**) $\sigma_{3}=4$ MPa; (**c**) $\sigma_{3}=6$ MPa.

Figure 12 illustrates the relationships between energy and axial strain *ε*_1_ for the calcareous mudstone. The evolution of dissipative energy, elastic strain energy, and total energy is characterized by significant stages. Analyzing the characteristics of each stage can help reveal the mechanism of rock compression damage and failure.

1. The crack compression stage (OA): During the initial stage of axial stress loading, most of the deformation is attributed to microcrack compression and closure. The energy density in this stage changes minimally with a slow growth rate, resulting in low energy conversion efficiency. The energy in this phase is predominantly governed by $U_{e}$, with the total energy stored as elastic strain energy exceeding the energy dissipated in microcrack closure and grain friction.
2. The elastic deformation stage (AB): The microcracks are further compressed and closed, the curves of the $U_{e}$ and $U_{1}$ have the same tendency to grow faster, and the changes in the $U_{d}$ and $U_{3}$ are very small. The total energy stored during this phase is primarily elastic strain energy.
3. The crack stable propagation stage (BC): Microcracks in the rock initiate and propagate, leading to plastic deformation. The rates of $U_{e}$ and $U_{1}$ continue to increase rapidly, while the growth rate of $U_{d}$ and $U_{3}$ accelerates.
4. The crack unstable propagation stage (CD): Cracks extend and develop in the rock unstably, with $U_{e}$ and $U_{1}$ continue to increase. However, the rate of increase in $U_{e}$ decreases. Additionally, as the rate increases, the changes in $U_{d}$ and $U_{3}$ become more noticeable, indicating accelerated crack expansion and penetration in the rock, accompanied by increased damage and plastic deformation.
5. The post-peak failure stage (DE): Microcracks coalesce and connect to generate a macro-failure plane at the peak stress, leading to rock fails. During this stage, the increase in $U_{1}$ occurs at a slower rate. There is a reversal of $U_{e}$, where stored $U_{e}$ is converted and released, resulting in sharp changes in $U_{d}$ and $U_{3}$. Most of the energy is dissipated as macro-fractures develop and deform.

**Figure 13:**
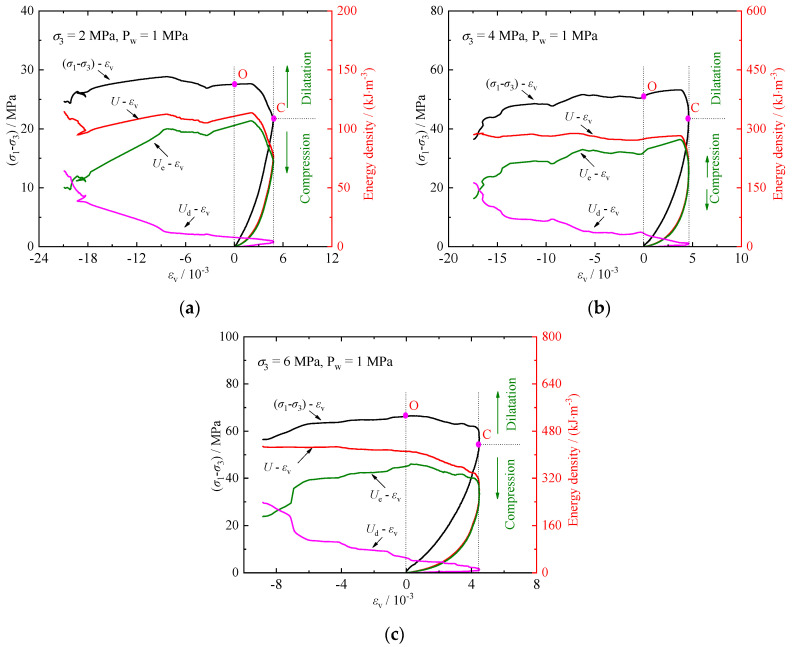
Evolutions of energy-volumetric strain: (**a**) $\sigma_{3}=2$ MPa; (**b**) $\sigma_{3}=4$ MPa; (**c**) $\sigma_{3}=6$ MPa.

Volumetric strain can effectively characterize energy change during the rock loading process. The energy and volumetric strain curves exhibit similar evolutionary laws during loading (Figure 13). Before reaching the damage stress (point C), the rock is in the compression phase. $U_{e}$ and U exhibit similar trends, with only a small difference between them, and changes in $U_{d}$ are minimal. After loading to the damage stress, the rock enters the dilatation phase, with the trend of $U_{e}$ and U is diverging, and the difference between them increasing, while $U_{d}$ increases sharply.

As the axial load increases, the elastic strain energy stored within the calcareous mudstone gradually accumulates. Concurrently, cracks within the calcareous mudstone are generated, extended, penetrated, and slipped, resulting in an increase in dissipated energy and leading to plastic deformation and damage accumulation. This process gradually decreases the strength of the calcareous mudstone and ultimately causes its complete loss of strength.

### 3.4. Failure Characteristics

The evolution of energy during each stage of the compressive failure process of calcareous mudstones can lead to variations in the microstructure and permeability within the rock. These differences manifest at the macroscale through distinct failure characteristics of the rock.

**Figure 14:**
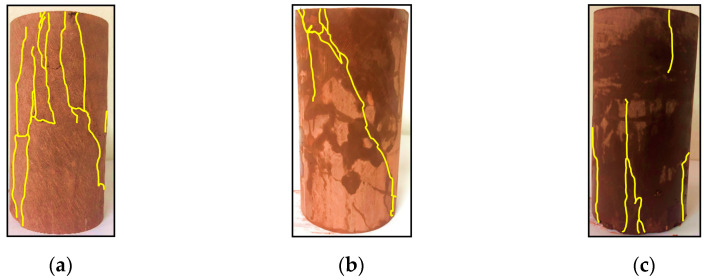
Failure patterns of the specimens at fluid pressure of 1 MPa: (**a**) $\sigma_{3}=2$ MPa; (**b**) $\sigma_{3}=4$ MPa; (**c**) $\sigma_{3}=6$ MPa.

The failure behavior of calcareous mudstone specimens is influenced by changes in confining pressure. Figure 14 shows the failure patterns of the rock specimens under a fluid pressure of 1 MPa. At a confining pressure of 2 MPa, the failed specimen displays combined shear-tensile fracture planes and exhibits a higher density of cracks. Under 4 MPa of confining pressure, the specimen demonstrates a shear fracture plane along with multiple cracks. At 6 MPa of confining pressure, the calcareous mudstone specimen presents multiple fracture planes.

**Figure 15:**
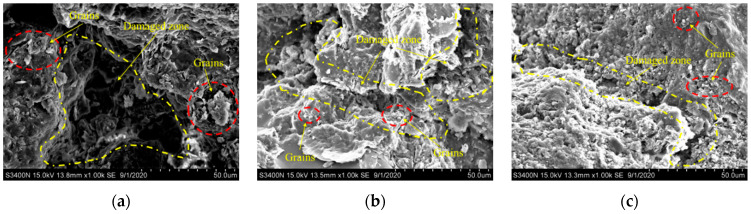
SEM images of microcracks in the specimens: (**a**) $\sigma_{3}=2$ MPa; (**b**) $\sigma_{3}=4$ MPa; (**c**) $\sigma_{3}=6$ MPa.

**Figure 16:**
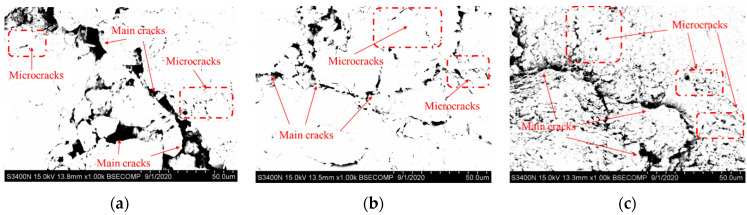
BSE images of microcracks in the specimens: (**a**) $\sigma_{3}=2$ MPa; (**b**) $\sigma_{3}=4$ MPa; (**c**) $\sigma_{3}=6$ MPa.

The post-experimental fracture surface micromorphology of the calcareous mudstone specimens is observed using SEM and backscattered electron (BSE) imaging at a magnification level of 1000. SEM images of the fracture surfaces of the failed calcareous mudstone specimens are shown in Figure 15. At confining pressures of 2 MPa and 4 MPa, the stripped mineral grains near the rock fracture are larger, and the cracks exhibit greater width and depth. Conversely, at a confining pressure of 6 MPa, the opposite trend is observed. BSE images of the fracture surfaces of the failed calcareous mudstone specimens are shown in Figure 16. The main cracks and microcracks demonstrate reduced expansion and width with increasing confining pressure.

In general, confining pressure restrains the initiation and propagation of microcracks within the calcareous mudstone, limiting the expansion of seepage paths. This phenomenon influences the evolution of its seepage behavior and enhances the strength of the calcareous mudstone specimens.

### 3.5. Mechanisms Analysis of Deformation Failure, Permeability, and Energy Evolution

Deformation and failure of mudstone are intricately linked to energy conversion, microstructural evolution, and changes in permeability. Mudstone is formed from weakly cemented clays through moderate epigenesis [43], often leading to landslides and other geological disasters [44]. Water–rock interaction causes changes in the mudstone microstructure [44], such as the formation of inhomogeneous distributions of hydrated cracks, and in the stress state [45], including expansion stresses in clay minerals subjected to water. These interactions also deteriorate the mechanical strength of the mudstone [43,44,45]. Cohesion between mineral grains weakens, friction reduces, and the energy required for rock failure decreases, promoting the accumulation of damage energy. Pore water pressure induces inhomogeneous stresses that expand cracks in the calcareous mudstone, increasing the connectivity of the crack network and widening seepage paths [39]. This results in dissipated energy and eventual deterioration of the internal structure of the rock, leading to weakening of its physical and mechanical properties [46].

The energy state of calcareous mudstone is dictated by its microstructure, while its macro-failure characteristics are influenced by the accumulated and dissipated of strain energy. Axial loading promotes microcrack initiation and damage accumulation, requiring increased energy for crack propagation. Water–rock interaction can diminish cohesion and friction, leading to the accumulation of damage energy. Inhomogeneous stresses facilitate crack growth and frictional sliding, ultimately increasing the dissipation energy of calcareous mudstone.

The calcareous mudstone undergoes a process influenced by water–rock interaction, resulting in changes in mineral composition, content, grain size, and micromorphology. This process includes microcrack initiation and propagation, structural failure, and weakening of mechanical properties. It represents a progressive and dynamic evolution invariably associated with energy generation and transfer [46].

## 4. Conclusions

Triaxial compression tests were conducted on calcareous mudstone specimens from the CYWDP in China to investigate their deformation and failure properties, permeability characteristics, and energy evolution under different confining pressures.

1. The stress–strain relationship of calcareous mudstone under varying confining pressures exhibits five stages based on characteristic stresses. The evolution of cracks effectively illustrates the deformation and failure process of calcareous mudstone. With increasing confining pressure, the characteristic stresses of each stage linearly increase, resulting in enhanced strength and ductility of the rock. For example, the peak stress *σ*_c_ (*P*_w_ = 0) increases by 52.35% and 20.85% when the confining pressure *σ*_3_ increases from 2 MPa to 4 MPa and 6 MPa, respectively.
2. This study explores the hydro-mechanical coupling of water, stress, and rock in the loading of calcareous mudstone. It reveals that fluid pressure weakens the mechanical properties of calcareous mudstone. Specifically, when *σ*_3_ = 2 MPa, the values of *σ*_cc_, *σ*_ci_, *σ*_cd_, and *σ*_c_ are reduced by 21.60%, 31.37%, 30.70%, and 36.61%, respectively, due to the effect of a fluid pressure of 1 MPa. Additionally, the evolution pattern of permeability in relation to axial/volumetric strain demonstrates significant stage characteristics associated with crack development and characteristic stresses. Permeability decreases with increasing confining pressure.
3. The evolution of energy density and stress–strain during loading exhibits similar phase characteristics. The damage stress serves as the turning point of energy dissipation and marks the threshold for delineating stages of the energy density-volumetric strain relationship curve. As an illustrative example, the dissipative energy *U*_d_ is considered. The values of *U*_d_ at *σ*_cc_, *σ*_cd_, and *σ*_c_ for *σ*_3_ = 2 MPa are 2.02, 4.09, and 12.39 kJ/m^3^, respectively. In contrast, the values of *U*_d_ at *σ*_cc_, *σ*_cd_, and *σ*_c_ for *σ*_3_ = 6 MPa are 3.93, 10.31, and 42.51 kJ/m^3^, respectively. Higher confining pressure correspond to greater energy values at characteristic stresses, necessitating more energy for rock deformation and failure.
4. Calcareous mudstone specimens exhibit different macroscopic failure modes due to variations in confining pressure. Furthermore, SEM and BSE tests reveal that with increasing confining pressure, the size and width of cracks diminish, and the mineral grains on crack surfaces reduce in size. These findings serve as a valuable reference for comprehending the microcrack response mechanism of calcareous mudstone to variations in confining pressure under hydro-mechanical coupling.

## Figures and Tables

**Table 1:** Test results.

Sample No.	Confining Pressure (MPa)	Fluid Pressure (MPa)	Crack Damage Stress (MPa)	Peak Stress (MPa)	Peak Axial Strain (×10^−3^)
DZ-1	2	/	31.38	45.53	8.91
DZ-2	4	/	55.17	69.38	14.23
DZ-3	6	/	55.34	83.84	14.93
DZ-4	2	1	21.75	28.87	9.52
DZ-5	4	1	43.10	53.26	10.78
DZ-6	6	1	54.21	66.51	11.67

## Data Availability

Data are contained within this article.
